# Evaluation of selection strategies in dual-purpose and specialized breeding of indigenous chicken

**DOI:** 10.1016/j.psj.2024.103916

**Published:** 2024-05-29

**Authors:** Sophie Miyumo, Chrilukovian B. Wasike, Evans D. Ilatsia, Jörn Bennewitz, Mizeck G.G. Chagunda

**Affiliations:** ⁎ Department of Animal Breeding and Husbandry in the Tropics and Sub-tropics, University of Hohenheim, Stuttgart 70599, Germany; † Livestock Efficiency Enhancement group (LEEG), Department of Animal and Fisheries Sciences, Maseno University, Maseno, Kenya; ‡ Kenya Agricultural and Livestock Research Organization, Poultry Research Program, Naivasha 20117, Kenya; § Department of Animal Breeding and Genetics, University of Hohenheim, Stuttgart 70599, Germany

**Keywords:** indigenous chicken, selection strategy, dual-purpose breed, specialized breed

## Abstract

This study aimed to evaluate various selection strategies for adoption in dual-purpose (**ICD**), meat (**ICM**) and layer (**ICL**) breeding goals in indigenous chicken breeding programs. The ICM goal aimed to improve live weight (LW_12_), daily gain (**ADG**) and egg weight (**EW_12_**) or together with feed efficiency and antibody response. For the ICL goal, age at first egg (**AFE**) and egg number (**EN_12_**) or together with feed efficiency and antibody response were targeted. In the ICD goal, the objective was to improve LW_12_, ADG, AFE and EN_12_ or together with feed efficiency and antibody response. Highest total index responses of US$ 49.83, US$ 65.71, and US$ 37.90 were estimated in indices targeting only production traits in the ICD, ICM and ICL goals, respectively. Highest index accuracy estimates of 0.77 and 0.70 were observed in indices that considered production and feed-related traits in the ICD and ICL goals, respectively, while in the ICM goal, the highest estimate of 0.96 was observed in an index targeting only production traits. Inbreeding levels ranged from 0.60 to 1.14% across the various indices considered in the breeding goals. Targeting only production traits in the ICD, ICM and ICL goals required the least number of generations of selection of 7.46, 5.50, and 8.52, respectively, to achieve predefined gains. Generally, a strategy targeting only production traits in a goal was the most optimal but resulted to unfavorable correlated responses in feed efficiency and antibody response. Addition of feed efficiency or/and antibody response in a goal was, however, not attractive due to the decline in total index response and accuracy and increase in inbreeding levels and number of generations of selection. Considering the feed availability and disease challenges in the tropics, choice of including feed efficiency or/and antibody response in the ICD, ICM and ICL goals should depend on targeted production system, resource availability to support breeding activities and magnitude of correlated responses on these traits when not included in the goals.

## INTRODUCTION

Indigenous chickens (**IC**) have a unique niche among rural resource poor households in developing countries (FAO, 2019). This is attributed to their low investment requirements, scavenging ability, self-propagation capacity, and adaptability to harsh environmental conditions in the tropics (Alders et al., 2018). Besides, IC contributes over 40 and 60% of chicken eggs and meat produced in developing countries and serve as a source of wealth creation in rural households (Khobondo et al., 2014). Despite these advantages, IC still have low production potential which limits their contribution to the growing protein demands in developing countries (Magothe et al., 2012a). Genetic improvement of IC is among the strategic interventions aimed to meet the needs of resource-poor poultry producers in developing countries (FAO, 2019). Past improvement efforts through crossbreeding of IC with exotic breeds, however, presented challenges such as genotype-environment incompatibility, potential erosion of IC genetic resource and increased production costs for resource-poor farmers (Magothe et al., 2012b).

The IC population is considered a major reservoir of the chicken genome and an essential genetic resource for breeding and conserving the vast gene pool they represent (Ajayi, 2010). Therefore, establishing a breeding program to address the constraints in IC production is fundamental, for which a broader genetic base of germplasm is a pre-requisite. Recent studies showed that the IC population comprises of various ecotypes and genotypes that display wide range of genetic variability with respect to morphological, production and adaptation characteristics (Magothe et al., 2010; Ngeno et al., 2013; Miyumo et al., 2016; Miyumo et al., 2023a). Presence of genetic diversity in these attributes could be exploited to improve performance in IC through selective breeding within the population. Besides, within population selection would assist in maintaining the IC attributes valued by actors along the value chain, control their genetic erosion and contribute towards their conservation (Okeno et al., 2013). Breeding programs targeting IC populations have already been established in some developing countries such as Malawi (Gondwe, 2004), Ethiopia (Tadelle et al., 2013) and Bangladesh (Faruque et al., 2017).

In Kenya, a national chicken breeding program aimed to address the needs of smallholder farmers was recently implemented (Ilatsia et al., 2017). The goal of the breeding program was defined in-consultation with stakeholders along the IC value chain. Majority of IC farmers prefer a dual-purpose IC breed that can produce, survive and reproduce under low input systems (Okeno et al., 2011b). Consequently, based on marketable end-products, a breeding goal targeting simultaneous improvement of meat and egg production within the IC population to develop a dual-purpose breed for low input systems was adopted in the breeding program (Ilatsia et al., 2017). However, for IC marketers, output levels from dual-purpose chicken under low-input systems is considered sub-optimal to meet their expected net profit (Okeno et al., 2012a). The growing consumer demand for IC eggs and meat is also an indication that production levels of dual-purpose IC under low-input systems may not be able to meet and sustain future consumer demands (Okeno et al., 2013). Besides, market forces are dynamic and change over time and this has an implication on the breeding goal. Therefore, to also meet the needs of marketers, and anticipated future consumers’ demands and market changes, formulation of alternative breeding goals targeting specialized production in IC was also considered in the breeding program (Ilatsia et al., 2017). Similar to commercial chicken breeding programs, the specialized breeding goal involves development of layer breed and a meat breed within the IC population. To maximize performance in the specialized IC breeds, the goal targets IC producers that are willing to adopt a semi-intensive system or at least an intensive system.

Growth rate, market live weight, age at sexual maturity, egg number, fertility, feed efficiency and disease resistance are some of the traits identified of economic importance among IC farmers (Okeno et al., 2011b; Bekele et al., 2020). Therefore, depending on the breeding goal, optimal combination of these traits in a multi-trait index should be targeted to maximize selection response, accuracy and index efficiency, and control inbreeding levels. This is because factors such as heritability of traits and genetic correlations among traits included in a goal influence index parameters and consequently, the overall genetic merit of an individual (Falconer and Mackay, 1996). For example, adaptability and fertility traits in chicken have low heritability and are negatively correlated with production traits (Mookprom et al., 2017; Miyumo et al., 2023b). This implies that low genetic gains would be achieved in these traits while in production traits, the gains are likely to slow down and consequently, result to a lower total index response. In a breeding goal targeting egg production in a medium size IC ecotype in Tanzania, Lwelamira and Kifaro (2010) previously reported that addition of antibody response in an index reduced gains in egg number and egg weight by 2.7% and 2.5%, respectively, compared to index that only considered the production traits. Given that genetic parameters, selection methods and intensities, and weighting factors vary across populations and/or studies, it is critical to assess the impact of combining various traits of interest in a goal on index parameters for the population of interest (Falconer and Mackay, 1996).

The aim of the current study was to simulate and evaluate various selection strategies for adoption in dual-purpose and specialized breeding goals in indigenous chicken breeding programs. In this study, a dual-purpose IC breed represents a terminal product constituting both males and females utilized for meat production and egg production, respectively, and post-egg production, culled females are used for meat production. On the other hand, a specialized IC layer breed constitutes of females as the terminal product for egg production and a specialized IC meat breed constitutes of males as the terminal product for meat production.

## MATERIALS AND METHODS

### Defining the Breeding Goal

The three breeding goals adopted in the IC breeding program in Kenya were considered in this study (Ilatsia et al., 2017). These included a dual-purpose IC (**ICD**) goal targeting both egg and meat production which represent smallholder farmers’ preference, and meat IC (**ICM**) goal and layer IC (**ICL**) goal representing alternative breeding goals targeting specialized meat production and specialized egg production, respectively. Traits corresponding to those regarded of economic importance by actors along the IC value chain were considered in each of the breeding goals (Okeno et al., 2011b). In ICD goal, live weight at 12 wk of age (**LW_12_**), juvenile growth rate (**ADG**), age at first egg (**AFE**), cumulative egg number at 12 wk’ post-onset of lay (EN_12_), daily feed intake (**ADFI**) and antibody response (**NDV-IgG**) were considered. Traits in the ICM goal were BW_12_, ADG, daily egg weight 12 wk from onset of lay (EW_12_), ADFI and NDV-IgG. In ICL goal, the traits were similar to those in ICD goal but excluding BW_12_ and ADG.

Use of LW_12_, ADG, EW_12_, AFE, EN_12_ as selection criteria traits to represent production traits preferred by farmers was based on recommendations by Miyumo et al. (2023b). Daily feed intake was used as an indicator trait for ability to efficiently utilize available feed resources given that feed intake has a positive genetic correlation with feed efficiency (Miyumo et al., 2023b). In addition, NDV-IgG was used as indicator trait for response to vaccination against Newcastle disease (**NCD**). The trait was considered of importance because NCD is a major constraint in chicken production systems in Kenya due to the frequent outbreaks which result to massive production and economic losses (Apopo et al., 2020). An aggregate goal (*H*) for ICD, ICM and ICL goals was defined based on relative economic values and breeding values corresponding to traits of interest as presented in equations 1, (2), (3), respectively.

(1) $$H_{ICD}=\;v_{1}LW+\;v_{2}ADG+\;v_{3}AFE+\;v_{4}EN+\;v_{5}ADFI+\;v_{6}NDVIgG$$

(2) $$H_{ICM}=\;v_{1}LW+\;v_{2}ADG+\;v_{3}EW+\;v_{4}ADFI+\;v_{5}NDVIgG$$

(3) $$H_{ICL}=\;v_{1}AFE+\;v_{2}EN+\;v_{3}ADFI+\;v_{4}NDVIgG$$

Where *H* is aggregate breeding value for traits included in dual-purpose (**ICD**), meat (**ICM**) and layer (**ICL**) goals, *v_i_* is the economic value corresponding to i^th^ trait considered in each of the goals; LW is body weight at 12 weeks of age; ADG is average daily gain between 8 to 20 wk of age; AFE is age at first egg; EN is cumulative egg number at 12 wk’ post-onset of lay; EW is average daily egg weight measured for 12 wk from onset of lay; ADFI is average daily feed intake; NDV-IgG is specific antibodies binding to Newcastle disease virus.

### Selection Scenarios

Several selection scenarios were designed in each goal to examine the impact of varying traits in an index on genetic (direct and correlated) gains, total economic responses to selection, index accuracy and rate of inbreeding per generation using the selection index theory (Hazel, 1943). This involved targeting only production traits in a goal or together with feed-related traits or/and immune-related traits. Four selection scenarios were considered in each of the goals and are presented in Table 1.

**Table 1:** Selection index scenarios considered in the dual-purpose (ICD), meat (ICM) and layer (ICL) indigenous chicken breeding goals.

		Traits^2^												
Goal	Index^1^	LW_12_	ADG	AFE	EN_12_	EW_12_	ADFI*	NDV-IgG*	RFI*	KLH-IgM*	MBW	BW_AFE_	FER	HAT
ICD	ICD_1_	+	+	+	+	−	−	−	−	−	−	−	−	−
	ICD_2_	+	+	+	+	−	+	−	−	−	−	−	−	−
	ICD_3_	+	+	+	+	−	−	+	−	−	−	−	−	−
	ICD_4_	+	+	+	+	−	+	+	−	−	−	−	−	−
														
ICM	ICM_1_	+	+			+	−	−	−	−	−			
	ICM_2_	+	+			+	+	−	−	−	−			
	ICM_3_	+	+			+	−	+	−	−	−			
	ICM_4_	+	+			+	+	+	−	−	−			
														
ICL	ICL_1_			+	+	−	−	−	−	−		−		
	ICL_2_			+	+	−	+	−	−	−		−		
	ICL_3_			+	+	−	−	+	−	−		−		
	ICL_4_			+	+	−	+	+	−	−		−		

1 In each index + indicates a trait is included in the goal and index; - indicates a trait is not included in the goal but is included in the index.

2 LW_12_ is body weight at 12 wk of age; ADG is average daily gain between 8 and 20 wk of age; AFE is age at first egg; EN_12_ is cumulative number of eggs at 12 wk from onset of lay; EW_12_ is average egg weight measured for 12 wk from onset of lay; ADFI is average daily feed intake; NDV-IgG is specific antibodies binding to Newcastle disease virus antigen; RFI is residual feed intake; KLH-IgM is natural antibody binding to KLH antigen; MBW is metabolic weight estimated between 8 and 20 wk of age; BW_AFE_ is body weight at sexual maturity; FER is fertility rate; HAT is hatchability rate.

⁎ In ICD, both ADFI and RFI were measured from 9 wk of age to 12 wk post onset of lay while both NDV-IgG and KLH-IgG were obtained as an average of titers measured at 16 and 28 wk of age; In ICM, ADFI and RFI were measured between 9 and 20 wk of age while NDV-IgG and KLH-IgG were each measured at 16 wk of age; In ICL, ADFI and RFI were measured for 12 wk from onset of lay while NDV-IgG and KLH-IgG were each measured at 28 wk of age.

Desired-gain selection indices were also constructed in each of the goals according to the Yamada index to determine the impact of imposing restrictions in an index on genetic gains and number of generations required to achieve pre-defined gains (Yamada et al., 1975). This involved targeting only production traits in a goal or together with feed-related traits or/and immune-related traits while controlling for genetic change in traits not considered of economic importance but of biological significance and valued by farmers. Six selection scenarios based on Yamada Index were considered in each of the goals and are presented in Table 2.

**Table 2:** Restricted **s**election index scenarios considered in the dual-purpose (ICD), meat (ICM) and layer (ICL) indigenous chicken breeding goals.

		Traits^2^												
Goal	Index^1^	LW_12_	ADG	AFE	EN_12_	EW_12_	ADFI*	RFI*	NDV-IgG*	KLH-IgM*	MBW	BW_AFE_	FER	HAT
ICD	ICD_1_	+	+	+	+	0	−	−	−	−	0	0	0	0
	ICD_2_	+	+	+	+	0	+	−	−	−	0	0	0	0
	ICD_3_	+	+	+	+	0	−	+	−	−	0	0	0	0
	ICD_4_	+	+	+	+	0	+	+	+	−	0	0	0	0
	ICD_5_	+	+	+	+	0	+	+	−	+	0	0	0	0
	ICD_6_	+	+	+	+	0	+	+	+	+	0	0	0	0
														
ICM	ICM_1_	+	+			+	−	−	−	−	0			
	ICM_2_	+	+			+	+	−	−	−	0			
	ICM_3_	+	+			+	−	+	−	−	0			
	ICM_4_	+	+			+	+	+	+	−	0			
	ICM_5_	+	+			+	+	+	−	+	0			
	ICM_6_	+	+			+	+	+	+	+	0			
														
ICL	ICL_1_			+	+	0	−	−	−	−		0		
	ICL_2_			+	+	0	+	−	−	−		0		
	ICL_3_			+	+	0	−	+	−	−		0		
	ICL_4_			+	+	0	+	+	+	−		0		
	ICL_5_			+	+	0	+	+	−	+		0		
	ICL_6_			+	+	0	+	+	+	+		0		
														

1 In each index + indicates a trait is included in the goal and index; - indicates a trait is not included in the goal but is included in the index; 0 indicates a trait is included in the goal but held constant.

2 LW_12_ is body weight at 12 wk of age; ADG is average daily gain between 8 and 20 wk of age; AFE is age at first egg; EN_12_ is cumulative number of eggs at 12 wk from onset of lay; EW_12_ is average egg weight measured for 12 wk from onset of lay; ADFI is average daily feed intake; NDV-IgG is specific antibodies binding to Newcastle disease virus antigen; RFI is residual feed intake; KLH-IgM is natural antibody binding to KLH antigen; MBW is metabolic weight estimated between 8 and 20 wk of age; BW_AFE_ is body weight at sexual maturity; FER is fertility rate; HAT is hatchability rate.

⁎ In ICD, both ADFI and RFI were measured from 9 wk of age to 12 wk post onset of lay while both NDV-IgG and KLH-IgG were obtained as an average of titers measured at 16 and 28 wk of age; In ICM, ADFI and RFI were measured between 9 and 20 wk of age while NDV-IgG and KLH-IgG were each measured at 16 wk of age; In ICL, ADFI and RFI were measured for 12 wk from onset of lay while NDV-IgG and KLH-IgG were each measured at 28 wk of age.

### Phenotypic, Genetic, and Economic Parameters

Ethical approval to conduct this study was provided by the Institutional Animal Care and Use Committee (**IACUC**) of KALRO-Veterinary Science Research Institute (**VSRI**) (KALROVSRI/IACUC019/30082019).

Data on growth, egg, feed, immune and reproduction related traits recorded on a base population of 3,360 indigenous chicken individuals constituting 480 sires and 2,880 dams was used to estimate the genetic and phenotypic parameters. A series of bi-variate animal models as previously described by Miyumo et al. (2023b) were fitted to estimate genetic and phenotypic (co)variance components among traits by restricted maximum likelihood using the WOMBAT software (Meyer, 2007). Phenotypic variance, heritability, and genetic and phenotypic correlations between traits included in each index in the ICD, ICM, and ICL goals are presented in Tables 3, 4 and 5, respectively. Relative economic values for traits included in the ICD, ICM and ICL goals were obtained from previous estimates by Okeno et al. (2012b) and Mahoro (2017) and are presented in Tables 3, Table 4, Table 5, respectively.

**Table 3:** Relative economic values, phenotypic variance, heritability (diagonal), genetic (lower diagonal) and phenotypic (upper diagonal) correlation for traits considered in various selection scenarios in the dual-purpose (ICD) goal.

			Traits^1^												
Traits^1^	EV^2^	Vp^3^	LW_12_	ADG	AFE	EN_12_	EW_12_	ADFI	NDV-IgG	RFI	KLH-IgM	MBW	BW_AFE_	FERT	HAT
LW_12_	73.46	60978	**0.44**	0.47	−0.08	−0.05	0.10	0.44	0.05	0.36	0.04	0.82	0.64	0.00	0.00
ADG	18.46	6.00	0.43	**0.45**	0.18	−0.09	0.30	0.72	−0.18	−0.03	0.18	0.69	0.70	0.00	0.00
AFE	−13.94	372.00	−0.88	0.46	**0.31**	−0.20	0.28	0.04	−0.02	−0.06	−0.01	0.28	0.35	0.10	0.00
EN_12_	8.87	91.92	−0.17	−0.12	−0.54	**0.23**	−0.05	0.20	−0.02	−0.12	−0.07	−0.20	0.20	0.23	0.20
EW_12_	0.05	16.72	0.22	0.35	0.74	−0.61	**0.64**	0.33	−0.10	0.10	−0.10	0.28	0.18	0.07	−0.49
ADFI	−6.17	249.44	0.78	0.58	0.27	0.40	0.61	**0.37**	0.22	0.09	0.36	0.32	0.32	0.00	0.00
NDV-IgG	−18.31	1.61	0.06	−0.56	−0.42	−0.49	−0.37	0.65	**0.18**	0.14	0.25	0.10	0.10	0.05	0.00
RFI	-	224.89	−0.66	−0.47	0.35	−0.44	0.45	0.77	0.62	**0.36**	0.13	−0.05	−0.05	0.00	0.00
KLH-IgM	-	1.96	−0.47	−0.22	−0.39	−0.37	−0.43	0.51	−0.50	0.53	**0.30**	0.11	−0.08	0.00	0.00
MBW	-	270.17	0.75	0.86	0.25	0.72	0.24	0.48	0.23	−0.57	0.49	**0.32**	0.34	0.00	0.00
BW_AFE_	-	29368	0.62	0.77	0.46	−0.59	0.46	0.53	−0.35	0.06	−0.41	0.52	**0.33**	0.13	0.03
FERT	-	16.70	0.00	0.00	0.59	−0.23	−0.17	0.02	0.05	0.00	0.00	0.00	−0.12	**0.16**	0.02
HAT	-	18.10	0.00	0.00	0.00	−0.20	−0.49	0.00	0.00	0.00	0.00	0.00	0.08	0.43	**0.06**

1 LW_12_ is body weight at 12 wk of age (g); ADG is average daily gain between 8 and 20 wk of age (g/d); AFE is age at first egg (days); EN_12_ is cumulative number of eggs at 12 wk from onset of lay; EW_12_ is average egg weight measured for 12 wk from onset of lay (g/d); ADFI is average daily feed intake measured from 9 wk of age to 12 wk post onset of lay (g/d); NDV-IgG is an average of specific antibodies binding to Newcastle disease virus antigen measured at 16 and 28 wk of age (ng/mL); RFI is residual feed intake measured from 9 wk of age to 12 wk post onset of lay (g/d); KLH-IgM is an average of natural antibody binding to KLH antigen measured at 16 and 28 wk of age (ng/mL); MBW is metabolic weight estimated between 8 and 20 wk of age (g); BW_AFE_ is body weight at sexual maturity (g); FER is fertility rate (%); HAT is hatchability rate (%).

2 EV is economic values in US$

3 Vp is phenotypic variance

**Table 4:** Relative economic values, phenotypic variance, heritability (diagonal), genetic (lower diagonal) and phenotypic (upper diagonal) correlation for traits considered in various selection scenarios in the meat (ICM) goal.

			Traits^1^							
Traits^1^	EV^2^	Vp^3^	LW_12_	ADG	EW_12_	ADFI	NDV-IgG	RFI	KLH-IgM	MBW
LW_12_	73.46	60978	**0.44**	0.47	0.10	0.44	0.05	0.36	0.04	0.82
ADG	18.46	6.00	0.43	**0.45**	0.30	0.72	−0.18	−0.03	0.18	0.69
EW_12_	0.05	16.72	0.22	0.35	**0.64**	0.04	−0.04	0.01	−0.03	0.12
ADFI	−6.17	105.85	0.78	0.58	0.10	**0.36**	0.20	0.05	0.56	0.61
NDV-IgG	18.31	16.13	0.06	−0.56	−0.11	0.34	**0.10**	0.04	0.01	0.12
RFI	-	95.42	−0.66	−0.52	−0.13	0.60	0.58	**0.43**	0.02	−0.01
KLH-IgM	-	1.04	−0.47	−0.22	0.26	0.41	−0.55	0.62	**0.22**	0.27
MBW	-	270.17	0.75	0.86	0.05	0.44	0.49	−0.52	0.46	**0.32**

1 LW_12_ is body weight (g) at 12 wk of age; ADG is average daily gain (g/d) between 8 and 20 wk of age; EW_12_ is average egg weight (g/d) measured for 12 wk from onset of lay; ADFI is average daily feed intake (g/d) measured between 9 and 20 wk of age; NDV-IgG is specific antibodies binding to Newcastle disease virus (ng/mL) measured at 16 wk of age; RFI is residual feed intake (g/d) measured between 9 and 20 wk of age; KLH-IgM is natural antibody binding to KLH antigen (ng/mL) measured at 16 wk of age; MBW is metabolic weight (g) estimated between 8 and 20 wk of age.

2 EV is economic values in US $

3 Vp is phenotypic variance

**Table 5:** Relative economic values, phenotypic variance, heritability (diagonal), genetic (lower diagonal) and phenotypic (upper diagonal) correlation for traits considered in various selection scenarios in the layer (ICL) goal.

			Traits^1^							
Traits^1^	EV^2^	Vp^3^	AFE	EN_12_	EW_12_	ADFI	NDV-IgG	RFI	KLH-IgM	BW_AFE_
AFE	−13.94	372.00	**0.31**	−0.20	0.28	0.04	−0.02	−0.06	−0.01	0.31
EN_12_	8.87	91.92	−0.54	**0.23**	−0.05	0.11	−0.02	−0.12	−0.07	0.20
EW_12_	0.05	16.72	0.74	−0.61	**0.64**	0.13	−0.10	0.10	−0.10	0.18
ADFI	−6.17	210.00	0.27	0.40	0.61	**0.37**	0.03	0.89	0.04	0.32
NDV-IgG	18.31	6.01	−0.42	−0.49	−0.37	0.52	**0.25**	0.15	−0.16	0.10
RFI	-	191.31	0.35	−0.44	0.45	0.74	0.50	**0.30**	0.01	−0.05
KLH-IgM	-	2.42	−0.39	−0.49	−0.37	0.52	−0.49	0.33	**0.41**	−0.08
BW_AFE_	-	29368	0.46	−0.59	0.46	0.53	−0.35	0.06	−0.35	**0.33**

1 AFE is age at first egg (days); EN_12_ is cumulative number of eggs at week 12 from onset of lay; EW_12_ is average egg weight (g/d) measured for 12 wk from onset of lay; ADFI is average daily feed intake (g/d) measured 12 wk from onset of lay; NDV-IgG is specific antibodies binding to Newcastle disease virus (ng/mL) measured at 28 wk of age; RFI is residual feed intake (g/d) measured 12 wk from onset of lay; KLH-IgM is natural antibody binding to KLH antigen (ng/mL) measured at 28 wk of age; BW_AFE_ is body weight at sexual maturity (g).

2 EV is economic values in US $

3 Vp is phenotypic variance

### Population Structure and Derivation of Selection Intensities

The population structure and biological parameters used in this study are a representation of the IC nucleus flock in the breeding station at the Non-Ruminant Research Institute of the Kenya Agriculture and Livestock Research Organization (**NRI-KALRO**) (Ilatsia et al., 2017). This involves retaining the best 80 males and 480 females in each generation. A mating ratio of 1 male to 6 females is utilized to optimize on male fertility and mating frequency. This is because, initially, use of one male to 10 females as recommended by Woodard and Abplnanalp (1967) resulted to low fertility rates and by testing different mating ratios within the breeding program, reduction of number of females to 6 per male was found to be the most optimal. This was based on the improved fertility rates from 50 to 80%. Considering a single hatch, each female in this population lays on average 20 eggs per clutch. Taking into account fertility (80%) and hatchability (70%) rates, each female produces 11 progenies resulting to a total of 5,376 chicks. Subsequently, adjusting for mortality of 20% by the time birds are subjected to selection and mated results to a total of 4,300 birds. Assuming a 50:50 sex ratio, 2,150 birds are produced in each sex. Therefore, selecting the best 80 males and 480 females from a single hatch in each generation results to selection proportions of 3.7% and 22% for males and females, respectively. The selection proportions result to selection intensities of 2.175 and 1.346 for the males and females, respectively. This gives an average selection intensity of 1.76 which falls within the range of selection intensities typically used in commercial chicken breeding programs (Zerehdaran et al., 2009; Chomchuen et al., 2022). Best candidates are selected by truncation of breeding values (**EBVs**) estimated from best linear unbiased prediction (**BLUP**) and used as replacement stock in the nucleus. For sex-limited traits such as egg production, male candidates are selected based their female ancestors and sib performances while female candidates are selected based on own performance, and female ancestor and sib performances. Selection for growth, feed efficiency and immune related traits are based on own, ancestor and sib performances.

So far, 2 cycles of selection have been carried out within the study population. Being in the initial stages of implementation, the breeding program targets selection for BW_12_ in the ICM goal, AFE in the ICL goal and both traits in the ICD goal for simplicity and effectiveness. Genetic trends and standard deviations of breeding values for traits under selection in the study population across three generations in each of the goals are presented in Table 6. Genetic trends per generation were estimated using breeding values adjusted to mean performance in each generation.

**Table 6:** Genetic trends (± SD)^1^ for traits under selection in the dual-purpose (ICD), meat (ICM) and layer (ICL) indigenous chicken breeding goals in Kenya.

		Traits^3^					
Goal	Gen^2^	LW_12_ (g)			AFE (days)		
ICD		All	Males	Females	All	Males	Females
	G_0_	1066.24 ± 215.67	1083.84 ± 218.29	1052.88 ± 214.66	168.12 ± 2.77	168.14 ± 2.69	168.11 ± 2.81
	G_1_	1133.08 ± 206.42	1164.69 ± 194.61	1079.05 ± 215.52	167.54 ± 5.38	167.43 ± 5.37	167.60 ± 5.39
	G_2_	1206.74 ± 225.19	1253.49 ± 211.67	1134.08 ± 231.57	167.00 ± 5.16	167.01 ± 4.88	166.98 ± 5.37
ICM		All	Males	Females			
	G_0_	1087.65 ± 215.51	1095.08 ± 217.61	1084.36 ± 214.74			
	G_1_	1164.07 ± 206.35	1192.35 ± 195.09	1170.45 ± 215.05			
	G_2_	1268.12 ± 224.66	1283.60 ± 210.79	1250.46 ± 231.08			
ICL					All	Males	Females
	G_0_				167.07 ± 2.65	167.09 ± 2.61	167.06 ± 2.69
	G_1_				165.18 ± 2.64	164.95 ± 2.26	165.29 ± 2.80
	G_2_				164.71 ± 5.61	164.90 ± 5.23	164.63 ± 5.79

1 ± SD is standard deviation of estimated breeding values.

2 Gen is generation; G_0_ is base generation, G_1_ is generation one, G_2_ is generation two.

3 LW_12_ is body weight measured at 12 wk of age, AFE is age at first egg.

### Evaluation of the Selection Scenarios

Deterministic simulation was used to model the various selection index scenarios in each of the breeding goal using the SelAction program (Rutten et al., 2002). A 2-tier closed nucleus breeding structure was considered because it resembles what is being practiced in most developing countries such as the Horro breeding program in Ethiopia and the National Black Australorp breeding program in Malawi (Gondwe, 2004; Tadelle et al., 2013). Tier one constitutes of a nucleus in which activities involve phenotyping, estimating breeding values, selection and mating. Tier two constitutes of farmers that utilize culled flock from the nucleus for production. Selection indices (I) presented in Table 1 were computed by summing true breeding values for traits included in the breeding goals and their respective economic weights using equation 4 (Rutten et al., 2002).

(4) $$I=\;b_{1}^{\prime }X_{1}+b_{2}^{\prime }X_{2}+\cdot \cdot \cdot +b_{i}^{\prime }X_{i}$$

where I is total response for a selection index; b is *m x 1* vector of selection index weights for *m* traits included in the goal; X is *k x 1* vector of source of information (phenotypic records of the candidate and/or its relatives) for *k* traits in the goal and index.

The index weight (**b**) for each trait included in the goals were estimated using equation 5.

(5) $$b=\;P^{-1}Ga$$

where P is *k x k* phenotypic (co)variance matrix for the traits included in the selection index; G is *m x n* genetic (co)variance matrix for *m* traits in the goal and *n* correlated traits in the selection index incorporating the additive genetic relationships between sources of information; a is *m x 1* vector of relative economic weights for traits in the goal.

Predicted response to selection (△R) for trait m in a breeding goal was calculated using equation 6 and accuracy of selection (r_I,H_) with respect to each index across the breeding goals was obtained using equation 7 (Rutten et al., 2002).

(6) $$\Delta R=\frac{i\left(b^{\prime }G\right)}{\sqrt{b^{\prime }Ga}}$$

(7) $$r_{\left(I,H\right)}=\;\sqrt{b^{\prime }Ga/a^{\prime }Ca}$$

where i is selection intensity; b is *m x 1* vector of selection index weights from equation 5; G is *m x n* genetic (co)variance matrix for *m* traits in the goal and *n* correlated traits in the selection index incorporating the additive genetic relationships between sources of information; a is *m x 1* vector of relative economic weights for *m* traits in the goal; C is *m x m* genetic (co)variance matrix among *m* traits included in the goal.

The rate of inbreeding from each index scenario across the goals was calculated using (8), (9) (Rutten et al., 2002).

(8) $$E\left(r\right)=(\alpha +\;\beta \left(BV-\;\bar{BV}\right)$$

(9) $$\Delta F=\;\frac{1}{2}\;N*E{\left(r\right)}^{2}$$

where E(r) is expected contribution of an individual, α is contribution of an individual with an average breeding value; β accounts for the increase of the contribution of parents with higher breeding value implying that the parents with high breeding values will have more selected progenies; ∆F is rate of inbreeding; N is number of individuals selected as parents in each generation.

Restricted selection indices (Table 2) were estimated using equation 4, however, index weights (b) were obtained using equation 10 (Yamada et al., 1975).

(10) $$b={\left(G^{\prime }R\right)}^{-1}Q$$

Where *G* is *m x n* genetic (co)variance matrix for *m* traits in the goal and *n* correlated traits in the index; R is *k x k* matrix of Wright's coefficient of relationship among individuals for *k* traits in the goal and index; Q is *m x 1* vector of desired gain for *m* traits in the goal.

Desired genetic gains (Q**-**vector) were calculated as the difference between desired and observed means (Lwelamira and Kifaro, 2010). Desired mean performances for goal were guided by previously defined desired gains by Ilatsia et al. (2017) for the IC population in Kenya. In the ICD goal, the breeding objective is to improve annual egg production from 80 eggs to 180 eggs (at least 3 clutches/year), reduce age at maturity from 168 d to 154 d and increase live weight at 12 wk of age from 1,054 g to 1,500 g in order to achieve at least 2,000 g of market weight at 26 wk of age. In addition, the goal aims to control genetic change in reproductive traits related to fertility and hatchability. In the ICM goal, the breeding objective is to improve live weight at 12 wk of age from 1,054 g to 1,800 g in order to achieve at least 2,500 g of market weight at 24 wk and increase egg weight from 45 g to 55 g. In the ICL goal, the breeding objective is to reduce age at maturity from 168 d to 140 d with minimal impact on body weight at maturity and increase annual egg production from 80 to 280 eggs (at least 4 clutches/year). For feed efficiency and immune traits, the desired levels were within the capacity of the IC population as shown by performance of some individuals under intensive system (Miyumo et al., 2023b). Furthermore, the chosen mean levels were close to the performance of crosses between local and commercial exotic chicken (Peters et al., 2012; Yaman et al., 2020). Estimated mean performance for production, feed efficiency, antibody response and fertility traits in the base population (Table 7) were used as a starting point in this study. Intended improvements previously reported by Ilatsia et al. (2017) was used as a representation of desired genetic change for traits included in the various index scenarios (Table 2) and are presented in Table 7.

**Table 7:** Observed mean, desired mean and desired change for traits included in meat (ICM), layer (ICL) and dual-purpose (ICD) goals.

Goal	Trait^1^	Unit	Observed mean	Desired mean	Desired change	Percent change (%)
ICD	LW_12_	g	1054.25	1500.00	445.75	42.28
	ADG	g/d	8.52	9.61	1.09	12.80
	AFE	Days	168.00	154.00	−14.00	−8.33
	EN_12_	Number	52.00	60.00	8.00	15.38
	ADFI	g/d	105.19	100.69	−4.50	−4.28
	RFI	g/g	0.31	0.01	−0.30	−96.77
	NDV-IgG	ng/mL	8.85	11.00	2.15	24.29
	KLH-IgM	ng/mL	9.71	11.01	1.30	13.40
	MBW	g	230.05	230.05	0.00	0.00
	BW_AFE_	g	1416.81	1416.81	0.00	0.00
	EW_12_	g/d	47.86	47.86	0.00	0.00
	FERT	%	81.90	81.90	0.00	0.00
	HAT	%	58.20	58.20	0.00	0.00
ICM	LW_12_	g	1054.25	1800	745.75	70.74
	ADG	g/d	13.82	16.00	2.18	15.77
	EW_12_	g	47.86	55	7.14	14.92
	ADFI	g/d	95.16	90.00	−5.16	−5.42
	RFI	g/g	0.25	0.00	−0.25	−100.00
	NDV-IgG	ng/mL	6.48	10.00	3.52	54.32
	KLH-IgM	ng/mL	7.08	10.00	2.92	41.24
	MBW	g	188.43	188.43	0.00	0.00
						
ICL	AFE	Days	168.00	140.00	−28.00	−16.67
	EN_12_	Number	52.00	70.00	18.00	34.62
	ADFI	g/d	115.21	110.00	−5.21	−4.52
	RFI	g/g	0.37	0.00	−0.37	−100.00
	NDV-IgG	ng/mL	11.22	15.00	3.78	33.69
	KLH-IgM	ng/mL	12.33	15.00	2.67	21.65
	BW_AFE_	g	1416.81	1416.81	0.00	0.00
	EW_12_	g/d	47.86	47.86	0.00	0.00

1 LW_12_ is body weight at 12 wk of age; ADG is average daily gain between 8 and 20 wk of age; AFE is age at first egg; EN_12_ is cumulative number of eggs at 12 wk from onset of lay; EW_12_ is average egg weight measured for 12 wk from onset of lay; ADFI is average daily feed intake; NDV-IgG is specific antibodies binding to Newcastle disease virus antigen; RFI is residual feed intake; KLH-IgM is natural antibody binding to KLH antigen; MBW is metabolic weight estimated between 8 and 20 wk of age; BW_AFE_ is body weight at sexual maturity; FER is fertility rate; HAT is hatchability rate. *In ICD, both ADFI and RFI were measured from 9 wk of age to 12 wk post onset of lay while both NDV-IgG and KLH-IgG were obtained as an average of titers measured at 16 and 28 wk of age; In ICM, ADFI and RFI were measured between 9 and 20 wk of age while NDV-IgG and KLH-IgG were each measured at 16 wk of age; In ICL, ADFI and RFI were measured for 12 wk from onset of lay while NDV-IgG and KLH-IgG were each measured at 28 wk of age.

Expected genetic change per generation for *k* traits considered in each selection index (Table 7) was estimated using equation 11 (Yamada et al., 1975). This included direct responses for *m* traits included in the goal and correlated responses for *n* traits excluded from the goal.

(11) $$\Delta G^{*}=\;\frac{i_{I}}{\sigma_{I}}\;.\;b^{\prime }RG^{*}$$

Where ∆G* is *k x 1* vector of genetic gains per generation in *k* traits in an index; *i_I_* is selection intensity; *σ_I_* is standard deviation of an index; b is *m x 1* vector of index weights for *m* traits in the goal; R is *k x k* matrix of Wright's coefficient of relationship among individuals for *k* traits in the goal and index; G* is *m x n* genetic (co)variance matrix for *m* traits in the goal and *n* correlated traits in the index. Index standard deviation (*σ_I_*) was estimated using equation 12.

(12) $$\sigma_{I}=\;\sqrt{\left(b{}^{\prime }Pb\right)\;}$$

Where b is *m x 1* vector of index weights for *m* traits in the goal; P is *k x k* phenotypic (co)variance matrix for the traits included in the selection index.

The number of generations (T) required to attain the pre-defined breeding objectives was estimated using equation 13 (Yamada et al., 1975).

(13) $$T=\;\frac{\sigma_{I}}{i_{I}}$$

Where T is the number of generations required to attain the desired genetic gains; *σ_I_* is the standard deviation of the index; *i_I_* is the intensity of selection based on the index. Matrix equations from the desired-selection indices were solved using the R Software (R core Team, 2022).

To determine the number of years required to achieve the desired gain, a generation interval of 13 mo was considered in this study (Lwelamira and Kifaro, 2010). This was based on the time required to carry out the following activities: mating, collection of eggs, incubation and hatching for 2 mo; rearing of birds from day old to sexual maturity for 6 mo; and recording of egg production traits for a period of 3 mo’ post on-set of lay for 5 mo (3 mo of egg data recording and 2 mo of reproduction).

## RESULTS

Genetic gains (and correlated responses) for individual traits, total index response, index accuracy and rate of inbreeding per generation for different selection strategies in the ICD, ICM, and ICL goals are presented in Tables 8, 9 and 10, respectively. In the ICD goal, an index targeting only production traits (LW_12_, ADG, AFE, and EN_12_) had the highest genetic gains in these traits and total index response, and the lowest rate of inbreeding. Conversely, the index was associated with unfavorable correlated responses in feed-related and antibody response traits. Addition of ADFI and NDV-IgG in the goal showed that genetic gains of -1.14 to -3.05 g/d and 0.25 to 0.60 ng/mL, respectively, could be achieved. However, this reduced the genetic gains in LW_12_, ADG, AFE and EN_12_ by 6 to 25%, 11 to 36%, 16 to 53% and 5 to 63 %, respectively. Similarly, total index response reduced by US$ 5.70 to 9.82 while inbreeding rate increased by 0.04 to 0.26%. Simultaneous addition of ADFI and NDV-IgG in the goal had the highest negative impact on production traits and the index parameters. On the other hand, comparing the impact of adding the traits separately in the goal showed that ADFI had a more negative impact on the production traits and the index parameters compared to NDV-IgG. Based on index accuracy, the highest estimate was observed in an index with both production traits and ADFI in the goal while the least estimate was observed in an index targeting only production traits in the goal. Generally, for traits (RFI, KLH-IgM, MBW, BW_AFE_, EW_12_, FERT and HAT) not included in the goal but considered in an index, unfavorable correlated responses were estimated. Apart from KLH-IgM, EW_12_, and FERT, the highest estimates of unfavorable correlated responses were observed in an index that targeted only production in the goal.

**Table 8:** Genetic gain for individual traits, total response, accuracy and rate of inbreeding per generation in the dual-purpose goal (ICD) across various selection indices.

Genetic gains	Index^1^			
Traits^2^	ICD_1_	ICD_2_	ICD_3_	ICD_4_
LW_12_ (g)	**(47.21)**	**(40.96)**	**(44.60)**	**(35.19)**
ADG (g/d)	**(0.45)**	**(0.32)**	**(0.40)**	**(0.29)**
AFE (days)	**(−1.67)**	**(−0.79)**	**(−0.99)**	**(−1.40)**
EN_12_ (number)	**(1.04)**	**(0.39)**	**(0.99)**	**(0.59)**
ADFI (g/d)	6.51	**(−3.05)**	4.34	**(−1.14)**
NDV-IgG (ng/mL)	−0.72	−0.89	**(0.60)**	**(0.25)**
RFI (g/d)	2.46	0.02	1.10	0.05
KLH-IgM (ng/mL)	−0.70	−0.82	−0.68	−0.74
MBW (g)	20.15	14.32	18.50	11.54
BW_AFE_ (g)	−15.23	−9.14	−12.00	−10.05
EW_12_ (g/d)	−0.85	−1.40	−1.11	−1.73
FERT (%)	−1.01	−0.72	−0.55	−1.23
HAT (%)	−0.53	−0.24	−0.36	−0.49
Total index response (US$/unit gain)	49.83	41.86	44.13	40.01
Index accuracy	0.68	0.77	0.70	0.72
Inbreeding (%)	0.75	0.79	0.94	1.01

1 Estimates in bold and parenthesis are direct response for traits included in the goal; Estimates not in parenthesis are correlated responses for traits not included in the goal but are in the index.

2 LW_12_ is body weight at 12 wk of age; ADG is average daily gain between 8 and 20 wk of age; AFE is age at first egg; EN_12_ is cumulative number of eggs at 12 wk from onset of lay; EW_12_ is average egg weight measured for 12 wk from onset of lay; ADFI is average daily feed intake measured from 9 wk of age to 12 wk post onset of lay; NDV-IgG is an average of specific antibodies binding to Newcastle disease virus antigen measured at 16 and 28 wk of age; RFI is residual feed intake measured from 9 wk of age to 12 wk post onset of lay; KLH-IgM is an average of natural antibody binding to KLH antigen measured at 16 and 28 wk of age; MBW is metabolic weight estimated between 8 and 20 wk of age; BW_AFE_ is body weight at sexual maturity; FER is fertility rate; HAT is hatchability rate.

**Table 9:** Genetic gain for individual traits, response to selection, accuracy of selection and rate of inbreeding per generation in the meat production goal (ICM) across various selection indices.

Genetic gains	Index^1^			
Traits^2^	ICM_1_	ICM_2_	ICM_3_	ICM_4_
LW_12_ (g)	**(62.08)**	**(55.22)**	**(58.71)**	**(50.74)**
ADG (g/d)	**(0.73)**	**(0.60)**	**(0.62)**	**(0.51)**
EW_12_ (g/d)	**(0.72)**	**(0.54)**	**(0.65)**	**(0.55)**
ADFI (g/d)	5.59	**(−2.30)**	3.01	**(−2.29)**
NDV-IgG (ng/mL)	−0.40	−0.49	**(0.23)**	**(0.14)**
RFI (g/d)	1.13	0.04	0.09	0.09
KLH-IgM (ng/mL)	−0.95	−1.01	−0.02	−0.05
MBW (g)	18.20	15.04	17.14	12.45
Total index response (US$/unit gain)	65.71	62.60	64.57	63.01
Index accuracy	0.96	0.85	0.80	0.83
Inbreeding (%)	0.60	0.62	0.90	0.92

1 Estimates in bold and parenthesis are direct response for traits included in the goal; Estimates not in parenthesis are correlated responses for traits not included in the goal but are in the index.

2 LW_12_ is body weight at 12 wk of age; ADG is average daily gain between 8 and 20 wk of age; EW_12_ is average egg weight measured for 12 wk from onset of lay; ADFI is average daily feed intake measured between 9 and 20 wk of age; NDV-IgG is specific antibodies binding to Newcastle disease virus antigen measured at 16 wk of age; RFI is residual feed intake measured between 9 and 20 wk of age; KLH-IgM is natural antibody binding to KLH antigen measured at 16 wk of age; MBW is metabolic weight estimated between 8 and 20 wk of age.

**Table 10:** Genetic gain for individual traits, response to selection, accuracy of selection and rate of inbreeding per generation in the egg production goal (ICL) across various selection indices.

Genetic gains	Index^1^			
Traits^2^	ICL_1_	ICL_2_	ICL_3_	ICL_4_
AFE (days)	**(−3.06)**	**(-2.08)**	**(−2.53)**	**(−1.59)**
EN_12_ (number)	**(2.89)**	**(1.43)**	**(1.95)**	**(1.01)**
ADFI (g/d)	3.74	**(−4.10)**	2.45	**(−3.10)**
NDV-IgG (ng/mL)	−0.65	−0.71	**(0.33)**	**(0.07)**
RFI (g/d)	3.78	−0.04	4.19	−0.09
KLH-IgM (ng/mL)	−0.45	−0.49	−0.43	−0.55
BW_AFE_ (g)	−35.00	−40.25	−39.49	−45.62
EW_12_ (g/d)	−1.80	−2.04	−1.86	−2.54
Total index response (US$/unit gain)	37.90	34.21	35.39	36.27
Index accuracy	0.57	0.70	0.52	0.62
Inbreeding (%)	0.73	0.86	0.98	1.14

1 Estimates in bold and parenthesis are direct response for traits included in the goal; Estimates not in parenthesis are correlated responses for traits not included in the goal but are in the index.

2 AFE is age at first egg; EN_12_ is cumulative number of eggs at 12 wk from onset of lay; ADFI is average daily feed intake measured for 12 wk post onset of lay; NDV-IgG is specific antibodies binding to Newcastle disease virus antigen measured at 28 wk of age; RFI is residual feed intake measured for 12 wk post onset of lay; KLH-IgM is natural antibody binding to KLH antigen measured at 28 wk of age; BW_AFE_ is body weight at sexual maturity; EW_12_ is average egg weight measured for 12 wk from onset of lay.

In the ICM goal, targeting selection on only production traits (LW_12_, ADG and EN_12_) resulted to the highest genetic gains in these traits, total index response and index accuracy. However, this index had unfavorable correlated responses in feed-related and antibody response traits. Inclusion of ADFI and NDV-IgG in the goal indicated that genetic gains of -2.90 to -2.30 g/d and 0.14 to 0.23 ng/mL, respectively, can be attained. On the contrary, addition of ADFI and NDV-IgG in the goal reduced the genetic gains in LW_12_, ADG and EW_12_ by 5 to 18%, 15 to 30%, and 9 to 24%, respectively. Besides, total index response and index accuracy also reduced by US$ 1.14 to 3.11 and 11 to 16%, respectively. Feed intake in the goal had a more negative impact on total index response while NDV-IgG in the goal had a higher negative influence on index accuracy. Apart from EW_12_, simultaneous addition of ADFI and NDV-IgG in the goal had the highest negative impact on production traits. With respect to rate of inbreeding per generation, the highest estimate was observed in an index with production, ADFI and NDV-IgG traits in the goal while the least estimate was observed in an index targeting only production traits in the goal. For RFI, KLH-IgM and MBW which were not included in the goal but considered in an index, unfavorable correlated responses were estimated in these traits.

In the ICL goal, the highest genetic gains in production traits and total index response, and the least estimate of inbreeding level were observed in an index that targeted only AFE and EN_12_ in the goal. The index was, however, associated with unfavorable correlated responses in feed-related and antibody response traits. On the other hand, addition of ADFI and NDV-IgG in the goal resulted to genetic gains of -3.10 to -4.10 g/d and 0.07 to 0.33 ng/mL, respectively, in feed-related and antibody response traits. Conversely, this reduced genetic gains in AFE and EN_12_ by 5 to 18%, and 9 to 24%, respectively and total index response by US$ 1.14 to 3.11 whereas rate of inbreeding increased by 0.04 to 0.26%. Simultaneous selection for both ADFI and NDV-IgG had the highest negative impact on genetic gains in egg production traits and rate of inbreeding while addition of only ADFI to the goal had the most negative effect on total index response. Based on index accuracy, the highest estimate was observed in an index with both egg production traits and ADFI in the goal while the least estimate was observed in an index targeting both egg production traits and NDV-IgG in the goal. For RFI, KLH-IgM and MBW which were not included in the goal but considered in an index, unfavorable correlated responses were estimated in these traits.

Direct and correlated genetic gains, and index efficiency estimated from restriction indices in the ICD, ICM and ICL goals are presented in Tables 11, 12 and 13, respectively. Restricting genetic change in MBW, BW_AFE_, EW_12_, FERT and HAT to zero in the ICD goal reduced the genetic gains in production, feed intake and antibody response traits compared to the estimate obtained in the unrestricted indices (Table 8). In the ICM goal, however, setting the genetic change in MBW to zero increased the genetic gains in the production, feed intake and antibody response traits compared to the estimates obtained in the unrestricted indices (Table 9). Similar to the ICD goal, genetic gains in production, feed intake and antibody response traits in the ICL goal reduced when genetic change in BW_AFE_ and EW_12_ were restricted to zero. The highest impact of these restrictions across the goals was observed in an index that considered production traits together with ADFI, RFI, KLH-IgM, and NDV-IgG while least impact was seen in an index with only production traits.

**Table 11:** Genetic gain for individual traits from restricted indices and number of generations required to attain desired gains across various selection strategies in the dual-purpose (ICD) breeding goal.

Genetic gains	Index^1^					
Traits^2^	ICD_1_	ICD_2_	ICD_3_	ICD_4_	ICD_5_	ICD_6_
LW_12_ (g)	**(64.70)**	**(54.83)**	**(60.73)**	**(49.20)**	**(54.83)**	**(43.23)**
ADG (g/d)	**(0.16)**	**(0.13)**	**(0.15)**	**(0.12)**	**(0.13)**	**(0.11)**
AFE (days)	**(−2.03)**	**(−1.72)**	**(−1.91)**	**(−1.55)**	**(−1.72)**	**(−1.36)**
EN_12_ (number)	**(1.16)**	**(0.98)**	**(1.09)**	**(0.88)**	**(0.98)**	**(0.78)**
ADFI (g/d)	4.86	**(−0.55)**	−0.20	**(−0.50)**	**(−0.55)**	**(−0.44)**
RFI (g/d)	2.51	0.12	**(−0.04)**	**(−0.03)**	**(−0.04)**	**(−0.03)**
NDV-IgG (ng/mL)	−0.35	−0.89	−0.42	**(0.24)**	−0.45	**(0.21)**
KLH-IgM (ng/mL)	−0.44	−0.94	−0.60	−0.58	**(0.16)**	**(0.13)**
MBW (g)	**(0.00)**	**(0.00)**	**(0.00)**	**(0.00)**	**(0.00)**	**(0.00)**
BW_AFE_ (g)	**(0.00)**	**(0.00)**	**(0.00)**	**(0.00)**	**(0.00)**	**(0.00)**
EW_12_ (g/d)	**(0.00)**	**(0.00)**	**(0.00)**	**(0.00)**	**(0.00)**	**(0.00)**
FERT (%)	**(0.00)**	**(0.00)**	**(0.00)**	**(0.00)**	**(0.00)**	**(0.00)**
HAT (%)	**(0.00)**	**(0.00)**	**(0.00)**	**(0.00)**	**(0.00)**	**(0.00)**
Generations	6.89	8.13	7.34	9.06	8.13	10.31
Years^3^	7.46	8.81	7.95	9.82	8.81	11.17

1 Estimates in bold and parenthesis are direct response for traits included in the goal; Estimates not in parenthesis are correlated responses for traits not included in the goal but are in the index.

2 LW_12_ is body weight at 12 wk of age; ADG is average daily gain between 8 and 20 wk of age; AFE is age at first egg; EN_12_ is cumulative number of eggs at 12 wk from onset of lay; EW_12_ is average egg weight measured for 12 wk from onset of lay; ADFI is average daily feed intake measured from 9 wk of age to 12 wk post onset of lay; NDV-IgG is an average of specific antibodies binding to Newcastle disease virus antigen measured at 16 and 28 wk of age; RFI is residual feed intake measured from 9 wk of age to 12 wk post onset of lay; KLH-IgM is an average of natural antibody binding to KLH antigen measured at 16 and 28 wk of age; MBW is metabolic weight estimated between 8 and 20 wk of age; BW_AFE_ is body weight at sexual maturity; FER is fertility rate; HAT is hatchability rate.

3 Number of years estimated based on a generation interval of 13 months.

**Table 12:** Genetic gain for individual traits from restricted indices and number of generations required to attain desired gains across various selection strategies in the meat (ICM) breeding goal.

Genetic gains	Index^1^					
Traits^2^	ICM_1_	ICM_2_	ICM_3_	ICM_4_	ICM_5_	ICM_6_
LW_12_ (g)	**(135.59)**	**(122.25)**	**(128.58)**	**(108.08)**	**(111.31)**	**(105.04)**
ADG (g/d)	**(0.40)**	**(0.36)**	**(0.38)**	**(0.32)**	**(0.33)**	**(0.31)**
EW_12_ (g/d)	**(1.30)**	**(1.17)**	**(1.23)**	**(1.03)**	**(1.07)**	**(1.01)**
ADFI (g/d)	2.05	**(−0.85)**	−0.15	**(−0.75)**	**(−0.77)**	**(−0.73)**
RFI (g/d)	1.52	0.06	**(−0.04)**	**(−0.04)**	**(−0.04)**	**(−0.04)**
NDV-IgG (ng/mL)	−0.22	−0.31	−0.20	**(0.51)**	−0.09	**(0.50)**
KLH-IgM (ng/mL)	−0.11	−0.19	−0.13	−0.24	**(0.44)**	**(0.41)**
MBW (g)	**(0.00)**	**(0.00)**	**(0.00)**	**(0.00)**	**(0.00)**	**(0.00)**
Generations	5.50	6.10	5.80	6.90	6.70	7.50
Years^3^	5.96	6.61	6.28	7.48	7.26	7.69

1 Estimates in bold and parenthesis are direct response for traits included in the goal; Estimates not in parenthesis are correlated responses for traits not included in the goal but are in the index.

2 LW_12_ is body weight at 12 wk of age; ADG is average daily gain between 8 and 20 wk of age; EW_12_ is average egg weight measured for 12 wk from onset of lay; ADFI is average daily feed intake measured between 9 and 20 wk of age; NDV-IgG is specific antibodies binding to Newcastle disease virus antigen measured at 16 wk of age; RFI is residual feed intake measured between 9 and 20 wk of age; KLH-IgM is natural antibody binding to KLH antigen measured at 16 wk of age; MBW is metabolic weight estimated between 8 and 20 wk of age.

3 Number of years estimated based on a generation interval of 13 months.

**Table 13:** Genetic gain for individual traits from restricted indices and number of generations required to attain desired gains across various selection strategies in the layer (ICL) breeding goal.

Genetic gains	Index^1^					
Traits^2^	ICL_1_	ICL_2_	ICL_3_	ICL_4_	ICL_5_	ICL_6_
AFE (days)	**(−3.56)**	**(−2.56)**	**(−3.06)**	**(−2.23)**	**(−2.45)**	**(−1.83)**
EN_12_ (number)	**(2.29)**	**(1.65)**	**(1.97)**	**(1.43)**	**(1.57)**	**(1.18)**
ADFI (g/d)	2.81	**(−0.48)**	−0.17	**(−0.42)**	**(−0.46)**	**(−0.34)**
RFI (g/d)	1.60	0.09	**(−0.04)**	**(−0.03)**	**(−0.03)**	**(−0.02)**
NDV-IgG (ng/mL)	−0.17	−0.37	−0.20	**(0.30)**	−0.19	**(0.25)**
KLH-IgM (ng/mL)	−0.04	−0.16	−0.09	−0.11	**(0.23)**	**(0.17)**
BW_AFE_ (g)	**(0.00)**	**(0.00)**	**(0.00)**	**(0.00)**	**(0.00)**	**(0.00)**
EW_12_ (g/d)	**(0.00)**	**(0.00)**	**(0.00)**	**(0.00)**	**(0.00)**	**(0.00)**
Generations	7.86	10.94	9.14	12.55	11.45	15.30
Years^3^	8.52	11.85	9.90	13.60	12.40	16.58

1 Estimates in bold and parenthesis are direct response for traits included in the goal; Estimates not in parenthesis are correlated responses for traits not included in the goal but are in the index.

2 AFE is age at first egg; EN_12_ is cumulative number of eggs at 12 wk from onset of lay; ADFI is average daily feed intake measured for 12 wk post onset of lay; NDV-IgG is specific antibodies binding to Newcastle disease virus antigen measured at 28 wk of age; RFI is residual feed intake measured for 12 wk post onset of lay; KLH-IgM is natural antibody binding to KLH antigen measured at 28 wk of age; BW_AFE_ is body weight at sexual maturity; EW_12_ is average egg weight measured for 12 wk from onset of lay.

3 Number of years estimated based on a generation interval of 13 months.

With respect to index efficiency, 6.89 to 10.31 generations, 5.50 to 7.10 generations and 7.86 to 15.30 generations would be required to achieve the pre-defined desired gains in the ICD, ICM and ICL goals, respectively. These estimates correspond to approximately 7 to 11 y, 6 to 7 y, and 9 to 17 y for the ICD, ICM and ICL goals, respectively. The least number of generations/years was estimated in an index that targeted only production traits in the ICD (LW_12_, ADG, AFE and EN_12_), ICM (LW_12_, ADG and EW_12_) and ICL (AFE and EN_12_) goals. In contrast, addition of ADFI, RFI, KLH-IgM, and NDV-IgG traits in an index with production traits resulted to the highest number of generations/years estimated in the ICD, ICM and ICL goals.

## DISCUSSIONS

This study designed and evaluated various selection strategies in order to determine the most optimal index that can be utilized in indigenous chicken breeding to develop dual-purpose (**ICD**) and specialized (meat - ICM and layer - ICL) breeds. Strategies targeting only production traits in the ICD (LW_12_, ADG, AFE, EN_12_), ICM (LW_12_, ADG, EW_12_) and ICL (AFE, EN_12_) goals had the highest total index response and required the least number of years to attain pre-defined gains. Simultaneous selection for production traits and feed intake resulted to higher index accuracy in the ICD and ICL goals while in the ICM goal highest accuracy was estimated in an index with only production traits. The lowest rate of inbreeding was observed in an index with only production traits in the ICD, ICM and ICL goals.

The genetic gains obtained under ICD imply that it is possible to simultaneously improve LW_12_, ADG, AFE and EN_12_ in an individual for the development of a dual-purpose breed. However, the antagonistic relationship between egg production and growth traits was evident in this study as estimated genetic responses in the ICD goal were lower compared to the alternative goals (ICM and ICL) targeting specialized production (Chomchuen et al., 2022). The impact of the antagonistic relationship between egg production and growth traits on genetic gains in the ICD goal reflects the negative pleiotropic nature of genes acting on these traits. This implies that an increase in growth rate would result to heavy birds at sexual maturity and consequently, reduce egg production and vice-versa. Therefore, it would be pertinent to consider optimal balancing of egg production and growth traits in the ICD goal, with respect to index weights, to control for the negative genetic correlation between the traits. This is because there is a possibility of placing more weight on one trait than the other, especially for highly heritable traits such as growth traits and hence, likely to result to bias. Similar observations on the antagonistic relationship between growth and egg production traits have been previously reported in indigenous chicken and commercial populations (Nwagu et al., 2007; Lwelamira and Kifaro, 2010). For instance, in an IC population in Tanzania, selection for body weight at 16 wk of age negatively impacted genetic gains in egg production while improved egg production negatively affected growth performance (Lwelamira and Kifaro, 2010).

Crossbreeding is suggested a suitable breeding scheme to optimize genetic gains in a dual-purpose goal targeting simultaneous improvement of egg production and growth traits (Bijma et al., 2001). For example, under ICD, Okeno et al. (2013) estimated higher genetic gains in a crossbreeding scheme compared to a pure-breeding scheme, and attributed this observation to use of a commercial exotic breed (as sire line) in a two-way crossbreeding with indigenous chicken (as dam line) and genetic advantage from heterosis. The two-way crossbreeding scheme represents the cockerel exchange program which has extensively been used in most developing countries including Kenya and has improved productivity in IC populations (Magothe et al., 2012b). However, it has not been successful at farm level due to the negative impacts of genotype by environment effect on performance because majority of IC producers utilize low-input systems (Khobondo et al., 2014). Besides, this strategy encourages indiscriminate crossbreeding at on-farm level and hence, threatens the diversity and adaptive attributes of the IC population (Bett et al., 2013). Alternatively, development of different lines for growth and egg production traits for crossbreeding within the IC population can be considered to maintain their diversity and adaptive potential to sub-optimal management conditions (Scholtz and Theunissen, 2010; Okeno et al., 2013).

In Kenya, as in most developing countries, IC are marketed for meat when their live body weight is at least above 2,000 g at ages between 24 and 26 wk (Bett et al., 2011; Natukunda et al., 2011). Therefore, body weight at 16 wk of age is suggested a suitable selection criterion trait to achieve higher market weights and attract relatively higher prices (Lwelamira and Kifaro, 2010; Okeno et al., 2013). Results from this study, however, indicated that live weight at 12 wk of age (LW_12_) together with juvenile ADG (8 to 20 wk of age) can also be utilized to achieve body weight of at most 1,800 g at 12 wk of age to meet the current market weight requirement of 2,500 g at 26 wk of age under intensive management (Ilatsia et al., 2017). This observation could be attributed to the high heritability estimated in LW_12_ and ADG (Table 4) in the study population, which implies that relatively high prediction accuracies was achieved in these traits and hence, the high genetic gains. Further, the positive genetic correlations estimated between LW_12_ and ADG (Table 4) indicates that the traits share a common genetic background and therefore, selection for either of the traits is expected to meet the targeted objective in the ICM goal. To this regard, use of only LW_12_ in the ICM selection criterion to improve growth rate could be a suitable strategy because this would enable earlier selection for body weight and hence, reduce generation interval, increase genetic progress, and minimize resources required for data recording at later ages. In addition, earlier selection for body weight could be utilized to reduce the age at market weight and consequently reduce production costs undertaken by producers to 26 wk of age, as well as, maintain meat qualities highly valued by consumers (Bett et al., 2011; Magothe et al., 2012b).

Egg weight (EW_12_) during the early laying period (12 wk post-onset of lay) was also considered a trait of interest in the ICM goal. This is because indigenous chicken generally lay small sized eggs (41 g to 46 g) and this has a consequential effect on early growth performance due to the positive genetic correlation between egg weight with hatch weight and growth rates during the brooding period (Ghorbani et al., 2012; Nicknafs et al., 2012). Therefore, selection for improved EW_12_ is expected to result higher hatch weight and early growth performance, and consequently, have a positive impact on subsequent growth performance. Despite the benefit of including egg weight in the ICM goal, this likely to extend the generation interval because an additional 20 wk after body weight measurement at 12 wk of age would be required to measure EW_12_ in potential parents. In this case, developing a line for egg weight and mating it to a younger parent population within the same generation selected for LW_12_ could be an alternative strategy to minimize the increase in generation interval in the ICM goal.

The positive genetic gains for egg production under ICL was expected because the goal targeted increased EN_12_ and reduced AFE. Besides, given that these traits were moderately heritable in the base population (Table 5) could also explain the observed genetic gains in egg production. This is based on the direct relationship between heritability (an indicator of accuracy of estimated breeding values) and genetic gain, which implies that an increase in magnitude of heritability is expected to improve gains, assuming selection intensity and generation interval are constant (Falconer and Mackay, 1996). Presence of negative genetic correlation between AFE and EN_12_ (Table 5) may also have been a contributing factor to this observation. This is because early maturing birds start laying at earlier age and consequently, likely to have a higher laying rate in the early period than late maturing birds. Such an association is considered favorable since it enables the traits to complement each other when combined in an index and hence, increases genetic gains in egg production. Genetic gains for EN_12_ and AFE obtained in this study were within the range of previously reported estimates in indigenous chicken in Tanzania and Rhode Island Red population in Nigeria (Nwagu et al., 2007; Lwelamira and Kifaro, 2010). Age at sexual maturity and cumulative egg number during the early laying period are suggested suitable selection criterion traits in a goal targeting development a layer breed (Wolc et al., 2007). This is because the traits enable early selection for egg production which has consequential benefits on the generation interval, genetic progress and resources required for data recording at later ages. However, due to physiological variations among the different laying periods, low genetic correlations between egg numbers measured in the early-lay period with mid- and late-lay periods were previously reported by Wolc et al. (2007) and Mookprom et al. (2017). This is an indication that egg production in the early-lay period may not accurately predict expected production at later ages. Therefore, it would be necessary to further consider correlated effects of selection for egg production during the early laying period traits on subsequent laying periods in the current study population.

Comparison between the different indices designed in each of the goals suggest that it would be genetically and economically rewarding to adopt an index targeting only production traits. However, the unfavorable correlated genetic responses in ADFI observed in this index across the goals imply that improved productivity would increase feed intake. This was expected because feed intake is positively correlated with production (Aggrey et al., 2010). Due to constraints related to feed costs and seasonal availability and quality of feed resources in IC production systems, optimal balance of improved productivity to available feed resources is pertinent (Miyumo et al., 2016). An attempt to reduce feed intake by including ADFI in a goal, however, reduced genetic gains in production traits and total index response by US$ 7.97, US$ 3.11, US$ 3.69 in the ICD, ICM and ICL goals, respectively, compared to an index targeting only production traits in a goal. Since it is desirable to reduce feed-related costs with minimal compromise on productivity, selection for feed efficiency traits, such as residual feed intake (RFI), that are relatively less independent of production traits would be more beneficial than selection for ADFI (Archer et al., 2002). The negative genetic gains estimated in RFI across the goals indicate that improved feed efficiency can be achieved by selecting individuals that consume what they require (RFI = 0) or less (RFI < 0) to meet their production and maintenance requirements (Aggrey and Rekaya, 2013; Yuan et al., 2015). However, the reduction in genetic gains in targeted production traits across the goals due to improved feed efficiency could be attributed to the positive and strong genetic correlation between RFI and ADFI (Aggrey et al., 2010). This is because with improved RFI, the level of feed intake is significantly reduced, and this indirectly influences production levels negatively. The impact of improved RFI on production traits was, however, lower compared to an index that considered ADFI in a goal.

Immune-related traits were also considered of importance in the ICD, ICM and ICL goals due to the impact of diseases on performance and economic losses in IC production systems (Okeno et al., 2011a; Rist et al., 2015). The positive genetic response for NDV-IgG and KLH-IgM observed in all the goals indicate a potential to increase specific antibodies against Newcastle disease and natural antibodies, respectively, within the IC population. This means that antibody response to vaccination against Newcastle disease and general disease resistance would be enhanced and therefore, improved immunity. However, with improved immune competence, total index response under ICD, ICM and ICL would reduce by US$ 5.7, US$ 1.14 and US$ 2.51, respectively. This is also corroborated by the lower genetic gains estimated in targeted production and feed efficiency traits compared to an index excluding the antibody traits. These observations could reflect the antagonistic genetic correlations between immune traits with production and feed-related traits, an indication that higher antibody response would reduce productivity and increase feed intake (Chomchuen et al., 2022; Miyumo et al., 2023b). Besides, considering that production and antibody response are resource demanding functions, biological trade-offs between the functions with respect to nutrient allocation is also likely to have contributed to these observations (Zerjal et al., 2021).

To control the negative impact of including antibody traits in a goal on total index response, adoption of an index targeting only production and feed-related traits across the goals would be ideal but sound health management, such as bio-security measures and regular vaccinations, at on-farm level would also need to be considered. Provision of such high standard health management practices is, however, possible under intensive or semi-intensive systems where resource availability is not a challenge compared to free-range system that rely on non-conventional methods to control diseases (Okeno et al., 2012a). Since majority of IC producers practice free-range systems and prefer a dual-purpose IC breed that can produce and survive under low-input management, a strategy targeting immune competence alongside production and feed efficiency traits in the ICD goal is desirable. In this case, balancing index weights on the traits could be utilized to minimize the impact of genetic trade-offs between antibody response with production and feed efficiency on selection response. On the other hand, given that specialized IC breeds are intended for producers willing to invest in improved health management, an index without antibody traits could be utilized to optimize on productivity with respect to available feed resources in the ICM and ICL goals.

Although an important egg production trait, egg weight (EW_12_) was not considered a trait of interest in the ICL and ICD goals in the current study. This is because eggs from indigenous chicken can be marketed at their current egg weight (45 g–48 g) as consumers in the Kenyan market are not willing to pay more for larger eggs (Bett et al., 2013). However, the negative correlated responses in EW_12_ estimated in the ICD and ICL goals implies that selection for early sexual maturity and high egg number would reduce egg weight (Magothe et al., 2010). Due to uncertainties in future markets, it would be desirable to control for genetic change in egg weight given the current population mean estimate for EW_12_ (47.86 g) is fairly within the range of the market requirement (Bett et al., 2013).

The positive correlated response in MBW under ICD and ICM indicate that genetic improvement in the goals would increase maintenance requirement in dual-purpose and meat breeds. This observation was expected because targeted traits such as LW_12_ and ADG in the goals are positively correlated to metabolic body weight (Miyumo et al., 2023b). Increase in MBW is, however, undesirable because of the significant influence of maintenance requirement on the efficiency of feed utilization compared to production traits (Aggrey and Rekaya, 2013). While it may be preferable to reduce MBW to optimize utilization of feed resources towards productivity, this would have negative implications on body composition which is an important trait in meat producing breeds. This is because higher MBW is associated with increased fat content in the body composition while low MBW results to higher percentage of leanness in the body composition (Aggrey et al., 2010). The current IC market in Kenya prefers meat that is juicy and tender, and these meat quality traits are positively influenced by increased fat content that enhances the water-holding capacity of meat (Bett et al., 2011; Oloo et al., 2018). Similarly, reduced MBW would negatively impact the maintenance function which plays a critical role on an individual's adaptive capacity and physiological balance to cope with stressors within its environment (Knap and Rauw, 2008). Therefore, maintaining the current metabolic body weight in this study population would be desired.

The negative correlated response in BW_AFE_ in the ICD and ICL goals imply that selection for early sexual maturity would reduce mature body weight and could be due to the positive genetic correlations between AFE and BW_AFE_ (Miyumo et al., 2023b). In laying chicken, body weight at maturity is used as an indicator trait for body condition because of its consequential impact on an individual's productive life during the laying period. Therefore, it is desirable to prevent negative effects of early sexual maturity on mature body weight. In indigenous chicken, it is desired that birds intended for egg production attain sexual maturity when their live body weight is within the range of 1,200 g to 1,500g (Lwelamira and Kifaro, 2010). The mean estimate for BW_AFE_ in the current study population (1,416 g) was within the desired range and therefore, it would be suitable to control genetic change in mature live weight in the ICD and ICL goal. On the other hand, the negative correlated responses estimated in FERT and HAT under ICD indicate that improved egg productivity would reduce fertility and hatchability. These observations could be attributed to the negative genetic correlations between egg production and reproductive traits (Mookprom et al., 2017; Chomchuen et al., 2022). Considering that smallholder farmers are interested in a dual-purpose chicken that can self-reproduce, it would be imperative to prevent reduction in fertility and hatchability in the ICD goal (Akbas and Takma, 2005; Okeno et al., 2011b).

Restricting genetic change in MBW, BW_AFE_, EW_12_, FERT and HAT to zero in the current study, however, had a negative impact on the targeted production, feed efficiency and antibody traits across the goals. This was expected due to control for genetic change in traits that are unfavorably correlated to production, feed efficiency and antibody traits (Savegnago et al., 2011; Miyumo et al., 2023b). Given that these traits have no direct impact on revenues, the choice of whether to restrict their genetic change (compromise on profitability) or monitor their correlated responses in every selection cycle (prevent negative implications on traits directly contributing to revenues) is dependent on the importance of these traits. This is with respect to their biological significance (MBW and BW_AFE_), market dynamics (EW_12_) and value to farmers (FERT and HAT) in indigenous chicken production systems.

Index accuracy is a key determinant of response to selection because it provides an indication of how close the estimated breeding value is to the true breeding value (Falconer and Mackay, 1996). The parameter is, however, dependent on the magnitude of heritability of traits considered in an index (Zhang et al., 2020). This could explain the high index accuracy estimated across the various selection strategies under ICM compared to ICD and ICL. The ICM goal targeted highly heritable traits, such as growth, feed intake and NDV-IgG measured during the juvenile period, and egg weight. On the other hand, in the ICL goal, moderate to lowly heritable traits such as EN_12_ and NDV-IgG measured post-maturity were targeted and hence, the lowest estimates of selection accuracy observed in this goal. Accuracy of selection in the ICD goal was in between those obtained in the ICM and ICL goals and could be due to combination of traits that have high heritability with moderate to lowly heritable traits in the goal. Despite the low estimates, the index accuracy under ICL and ICD improved with addition of ADFI in the goals. It is possible that the goals may have benefited from additional information from feed intake, a highly heritable trait that is strongly correlated to egg production (van der Klein et al., 2015). This is also confirmed by the lack of improvement in index accuracy in the ICM goal when ADFI was included in an index alongside highly heritable traits such as growth and egg weight in this goal.

Generally, all the breeding strategies across the three goals had higher rates of inbreeding than the recommended level of 0.1% in livestock, which is considered an optimal level to maintain for potential evolutionary processes (Franklin and Frankham, 1998). This may be explained by the low effective population size of 274.29 in the current study population, since inbreeding level is influenced by the number of individuals contributing to variation within a population (Wang et al., 2016). Besides, the effective population size in the current population was lower than the recommended size of 500 to 1000 for conservation in domesticated animal populations (Franklin and Frankham, 1998). On the other hand, Woolliams and Bijma (2000) reported that when traits of low heritability are included in an index and BLUP is used, information from relatives is given more weight which increases the possibility of relatedness and hence, higher inbreeding rate is expected. This observation is consistent with findings in the current study since strategies under ICD and ICL that included low to moderately heritable traits, such as EN_12_ and NDV-IgG, had highest rate of inbreeding per generation.

It may also be of interest to have prior knowledge on the number of generations of selection it would take to achieve desired market requirements in a goal. This is due to its implications on the resources needed to support breeding activities and besides, such information can assist in planning of breeding activities with respect to available resources (Lwelamira and Kifaro, 2010). Depending on the goal, number of generations of selection is influenced by the traits considered in an index with respect to heritability and pleiotropic nature between traits (Yamada et al., 1975). This is because the genetic parameters directly influence the rate of genetic response per generation. For instance, an index with traits of high heritability and are favorably correlated, the rate of genetic response is expected to be high and hence, achievement of a targeted goal is likely to take a shorter time (Yamada et al., 1975).

In this study, selection strategies targeting only production traits in an index would be the most attractive to adopt in the ICD, ICM and ICL goals due to the least number of generations of selection estimated in each of the goals. Given that the production traits (LW_12_, ADG, EW_12_, AFE and EN_12_) considered in the index were moderate to highly heritable and favorably correlated (apart from growth and egg production traits in the ICD goal) could explain the low number of generations (Miyumo et al., 2023b). However, the unfavorable correlated responses in ADFI, RFI, NDV-IgG and KLH-IgM from the index imply that with improved productivity the resulting breeds from each of the goals are likely to be feed inefficient and less adaptable to environmental stressors. This trend is undesirable for chicken intended to produce under IC production systems in developing countries because the systems experience fluctuations in feed availability and quality, high feed costs and frequent disease out-breaks (Okeno et al., 2011b; Miyumo et al., 2018). Moreover, implementation of such an index would not meet the requirements preferred by IC producers, especially the resource poor farmers.

Combination of feed efficiency and antibody traits with production traits in an index, on other hand, would require more generations of selection compared to an index with only production traits. For instance, inclusion of RFI in an index required an additional 0.49 y, 0.32 y, and 1.28 y in the ICD, ICM and ICL goals, respectively. Similarly, inclusion of NDV-IgG and KLH-IgM in an index required an additional 1.34 to 2.35 y, 1.30 to 1.52 y, and 3.88 to 5.08 y under ICD, ICM and ICL, respectively. An index that considered RFI, NDV-IgG and KLH-IgM simultaneously required an additional 3.71 y, 1.73 y and 8.06 y in the ICD, ICM and ICL goals, respectively. These observations may be attributed to the antagonistic genetic correlation between production traits and antibody response traits, and the impact of nutrient competition between production and immune functions on feed efficiency (Miyumo et al., 2023b). This is because with improved antibody response, genetic gain in production and feed efficiency traits reduced and hence, the increase in time required to achieve targeted goals in ICD, ICM and ICL. Targeting only production traits or together with antibody response in Tanzanian local chicken, Lwelamira and Kifaro (2010) observed a similar pattern in the number of generations of selection required to attain desired gains in meat and layer goals. However, the estimates from Lwelamira and Kifaro (2010) were lower compared to the current study and could be attributed to differences in desired gains resulting from variation in market requirements between Kenya and Tanzania. Besides, contrary to the population used in the current study, Lwelamira and Kifaro (2010) used an ecotype (*Kuchi* ecotype) that is considered larger than normal-sized IC ecotypes in the ICM goal and a medium-sized ecotype identified to have a potential for egg production in the ICL goal.

With respect to pre-defined desired genetic change, the choice of which selection strategy to adopt in the ICD, ICM, and ICL goals in IC breeding programs is subject to resource availability to support breeding activities and targeted production environment for the terminal breed. For instance, dual-purpose chicken are mostly preferred by smallholder farmers that utilize low-input production systems with minimal investments in feed provision and health management (Okeno et al., 2012a). Therefore, a selection strategy that targets simultaneous improvement of growth, egg production, feed efficiency and antibody response would be desirable in the ICD goal. However, this would be at the expense of an additional 3.71 y of selection compared to the most efficient index targeting only production traits. On the other hand, the specialized IC breeding goals considered in this study aimed to target producers that are willing to adopt a semi-intensive system or at least an intensive system (Ilatsia et al., 2017). Under these production systems, the objective is to optimize on efficient productivity due to the high (∼70%) contribution of feed provision on production costs while integrating routine vaccination and bio-security measures in their management practices to control for diseases (Okeno et al., 2012a). In this case, a selection strategy that targets simultaneous improvement of production and feed efficiency traits would be a suitable option to consider in the ICM and ICL goals. Besides, such an index would require slightly more time compared to an index with production, feed efficiency and antibody response traits simultaneously.

## CONCLUSIONS

Selection strategy targeting only production traits in the dual-purpose (ICD) goal, growth traits in the meat (ICM) goal and egg production traits in the layer (ICL) goal in an index was the most attractive across the goals. This is based on the highest total index response, lowest rate of inbreeding per generation, and least number of generations of selection estimated in the index. Apart from the ICM goal, the low accuracy levels observed in this index in the ICD and ICL goals, however, is likely to have a negative implication on the rate of genetic response per generation. Besides, the unfavorable correlated responses on feed- and immune-related traits estimated in the index across the goals is undesirable. Under the ICD goal, terminal birds are expected to perform in low input systems experiencing feed availability and diseases challenges. Therefore, a strategy targeting also feed efficiency and antibody traits alongside production traits would be relevant in the dual-purpose goal given the favorable genetic gains in these traits and improved accuracy observed in the index. However, this would be at the expense of reduced total index response, and increased inbreeding levels and number of generations of selection required to achieve pre-defined gains. On the other hand, since terminal specialized breeds from the ICM and ICL goals are expected to perform under high input systems or at least semi-intensive systems, it would be beneficial to consider an index targeting simultaneous improvement of production and feed efficiency traits. This is because such production systems aim to optimize on efficient production against limited feed resources while integrating effective health management practices. Furthermore, the strategy would have a minimal negative impact on total index response, inbreeding levels and number of years required to attain the desired genetic gains with improved index accuracy (only in the ICL goal). Generally, these results demonstrate the possibilities to develop either dual-purpose or specialized chicken breeds that are feed efficient and immune competent within the IC population. However, use of economic values estimated from previous studies as weighting factors in the ICD, ICM, and ICL goals limited this study from capturing the current production costs and revenues in IC production systems in the estimation of total index response and index accuracy. This is because inflation changes over time due to market forces and these changes have an impact on the market value of inputs and outputs in livestock production systems, and consequently on the expected total index response and index accuracy. Therefore, index parameters estimated in this study should be interpreted with caution while future studies in a similar area should aim to estimate economic values for traits of interest in a breeding goal to obtain accurate estimates of index parameters.

## DISCLOSURES

The authors declare no conflicts of interest.
